# Variation in Petal and Leaf Wax Deposition Affects Cuticular Transpiration in Cut Lily Flowers

**DOI:** 10.3389/fpls.2021.781987

**Published:** 2021-11-24

**Authors:** Guiping Cheng, Ling Wang, Hairong Wu, Xinfan Yu, Nan Zhang, Xiaorong Wan, Lihong He, Hua Huang

**Affiliations:** ^1^College of Agriculture and Biology, Zhongkai University of Agriculture and Engineering, Guangzhou, China; ^2^Institute of Fruit Tree Research, Guangdong Academy of Agricultural Sciences/Key Laboratory of South Subtropical Fruit Biology and Genetic Resource Utilization, Ministry of Agriculture and Rural Affairs/Guangdong Provincial Key Laboratory of Tropical and Subtropical Fruit Tree Research, Guangzhou, China; ^3^Sericultural & Agri-Food Research Institute, Guangdong Academy of Agricultural Sciences/Key Laboratory of Functional Foods, Ministry of Agriculture and Rural Affairs/Key Laboratory of Agricultural Products Processing, Guangzhou, China; ^4^Customs Technology Center of Guangzhou Customs District, Guangzhou, China

**Keywords:** lily flowers, cuticular wax, leaf, tepal, transpiration

## Abstract

The vase life of cut flowers is largely affected by post-harvest water loss. Cuticular wax is the primary barrier to uncontrolled water loss for aerial plant organs. Studies on leaf cuticular transpiration have been widely conducted; however, little is known about cuticular transpiration in flowers. Here, the cuticular transpiration rate and wax composition of three lily cultivars were determined. The minimum water conductance of tepal cuticles was higher at the green bud than open flower stage. Lily cuticular transpiration exhibited cultivar- and organ-specific differences, where transpiration from the tepals was higher than leaves and was higher in the ‘Huang Tianba’ than ‘Tiber’ cultivar. The overall wax coverage of the tepals was higher compared to that of the leaves. Very-long-chain aliphatics were the main wax constituents and were dominated by *n*-alkanes with carbon (C) chain lengths of C_27_ and C_29_, and C_29_ and C_31_ in the tepal and leaf waxes, respectively. Primary alcohols and fatty acids as well as small amounts of alkyl esters, ketones, and branched or unsaturated *n*-alkanes were also detected in both tepal and leaf waxes, depending on the cultivar and organ. In addition, the chain-length distributions were similar between compound classes within cultivars, whereas the predominant C-chain lengths were substantially different between organs. This suggests that the less effective transpiration barrier provided by the tepal waxes may result from the shorter C-chain aliphatics in the tepal cuticle, compared to those in the leaf cuticle. These findings provide further insights to support the exploration of potential techniques for extending the shelf life of cut flowers based on cuticular transpiration barrier properties.

## Introduction

The surfaces of aerial plant organs are covered by cuticle, and its primary function is to prevent uncontrolled water loss (Riederer and Schreiber, 2001). Plant cuticle is predominantly a mixture of waxes, including very-long-chain fatty acids and their derivatives, cyclic terpenoids, with cutin polymers providing a scaffold matrix, mainly C_16_ and C_18_ with or without hydroxy groups (Yeats and Rose, 2013). It is well known that the cuticular waxes, rather than the cutin matrix, are the barrier to non-stomatal water loss (Riederer and Schreiber, 1995; Jetter and Riederer, 2016). The diversity of cuticular wax components and their spatial arrangements results in a wide variety of cuticular transpiration barrier properties across species, organs, and developmental stages (Jetter and Riederer, 2016; Huang et al., 2017; Bourgault et al., 2020). Previous studies have focused on how the barrier properties of leaf or fruit cuticles affect water loss or chemical substrates (Riederer and Schreiber, 2001; Jetter and Riederer, 2016), whereas few studies have been conducted on the barrier properties of flower cuticles.

The thin cuticle covering the petal surface plays an important role in interactions with pollinators (Whitney et al., 2011). Previous studies reported that cuticular transpiration in fruit cuticular membranes was higher than that of leaf cuticles. It implied that it might not be necessary to form such efficient cuticular barriers to transpiration for fruit as the life time is usually shorter than leaf tissues (Schreiber and Riederer, 1996). Chemical analysis has indicated that the wax layer of *Cosmos bipinnatus* var. ‘Sensation Pinkie’ petals contains relatively high concentrations of C_22_ and C_24_ fatty acids and primary alcohols, which are much shorter than those in the wax layer of leaves. Therefore, the petal cuticle exhibits weaker transpiration barrier properties than the leaf cuticles (Buschhaus et al., 2015). Similarly, C-chain lengths of waxes in petal cuticles of ‘Movie star’ and ‘Tineke’ roses were generally between C_26_ and C_29,_ but ranged from C_29_ to C_33_ in the leaf cuticles. The C-chain differences were thought to be one of the factors inducing higher transpiration rate for the petals compared to that for the leaves in roses (Cheng et al., 2019). A comparative study on cuticular wax chemical differences between flowers and other organs has also been conducted (Guo and Jetter, 2017). In addition, significant changes in the composition of the wax on snapdragon petals were found over 12days from the opening stage to senescence (Goodwin et al., 2003). However, more information about the chemical composition of petal cuticles and its contribution to barrier properties during petal development are still awaiting to be explored.

Oriental lily, a *Lilium* spp., is one of the most popular ornamental plants. Lilies are widely grown in temperate, subtropical, and tropical regions, and blooming from spring to early autumn. Oriental lily cut flowers usually comprise the flower head and the stem and keep some leaves. The lily flower head has two whorls of tepals (floral leaves) and each of the inner and outer whorls normally has three tepals (van Doorn and Han, 2011). The inner tepals are covered by the outer tepals during flower development. Lilies are widely planted by consumers for their elegant look and wide open, brightly colored tepals. The vase life of lily flowers varies with cultivar, cultivation, and post-harvest conditions. In addition, the shelf life of the cut flowers is impacted by rapid water loss or the deterioration of stem tissues after harvest (Torre and Fjeld, 2001; Cheng et al., 2020).

The present study aimed to characterize and compare the chemical composition of the cuticular waxes of lily tepals and leaves as well as to determine their potential contributions to limit cuticular transpiration from the tepals and leaves of three lily cultivars. The transpiration rate and chemical composition of the cuticular waxes (1) on the tepals at the green bud and open stages of the *Lilium* spp. cultivar ‘Casa Blanca’, and (2) on the leaves and tepals of two *Lilium* spp. cultivars ‘Huang Tianba’ and ‘Tiber’ were determined. These findings enabled comparisons of the cuticular wax chemicals on tepals at different development stages and organs, as well as link the transpiration barrier differences.

## Materials and Methods

### Plant Materials

**Figure 1:**
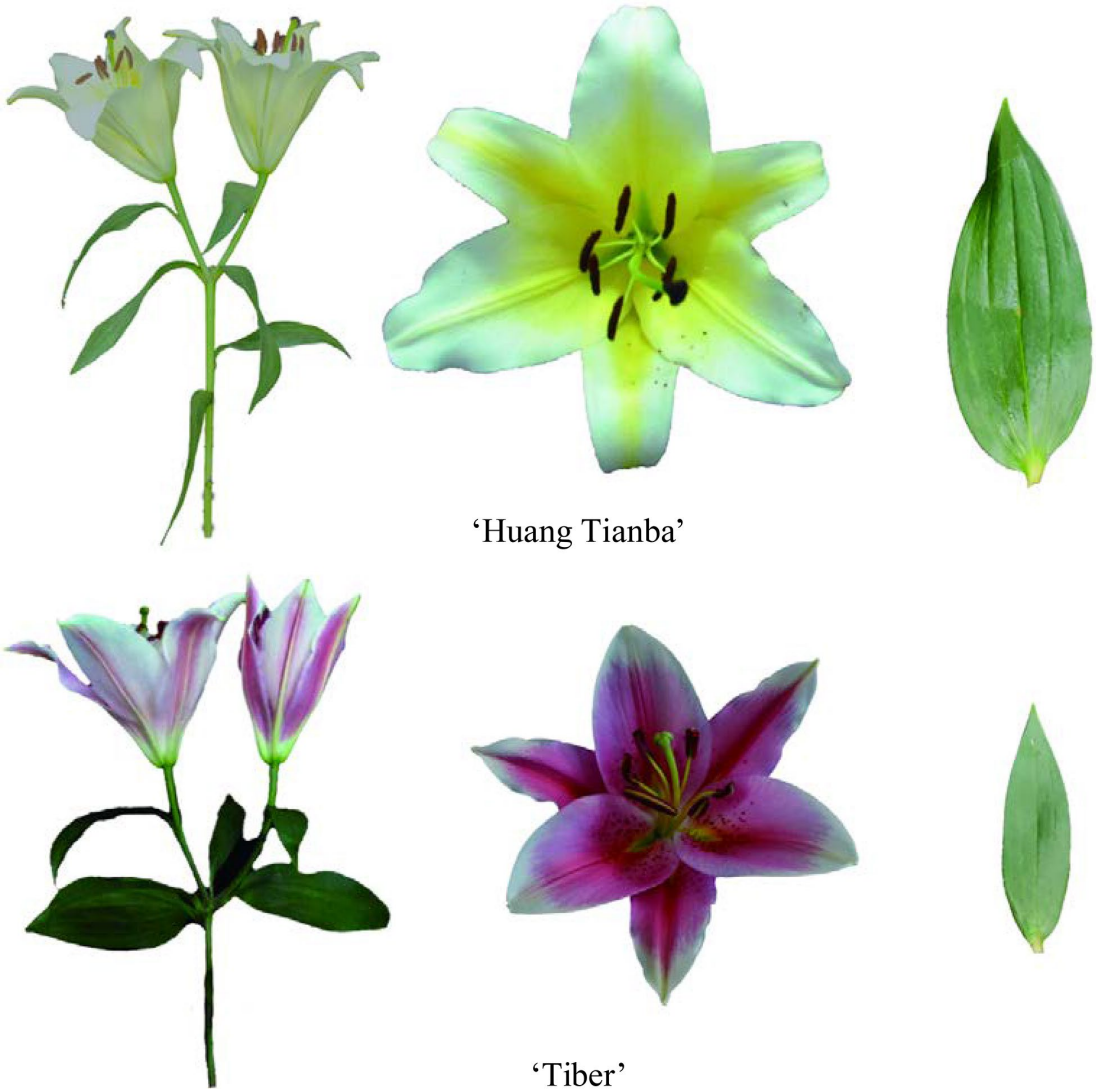
*Lilium* spp. (lily) flowers of the cultivars ‘Huang Tianba’ and ‘Tiber’. The cut flowers comprise open tepals, leaves, and stems.

**Figure 2:**
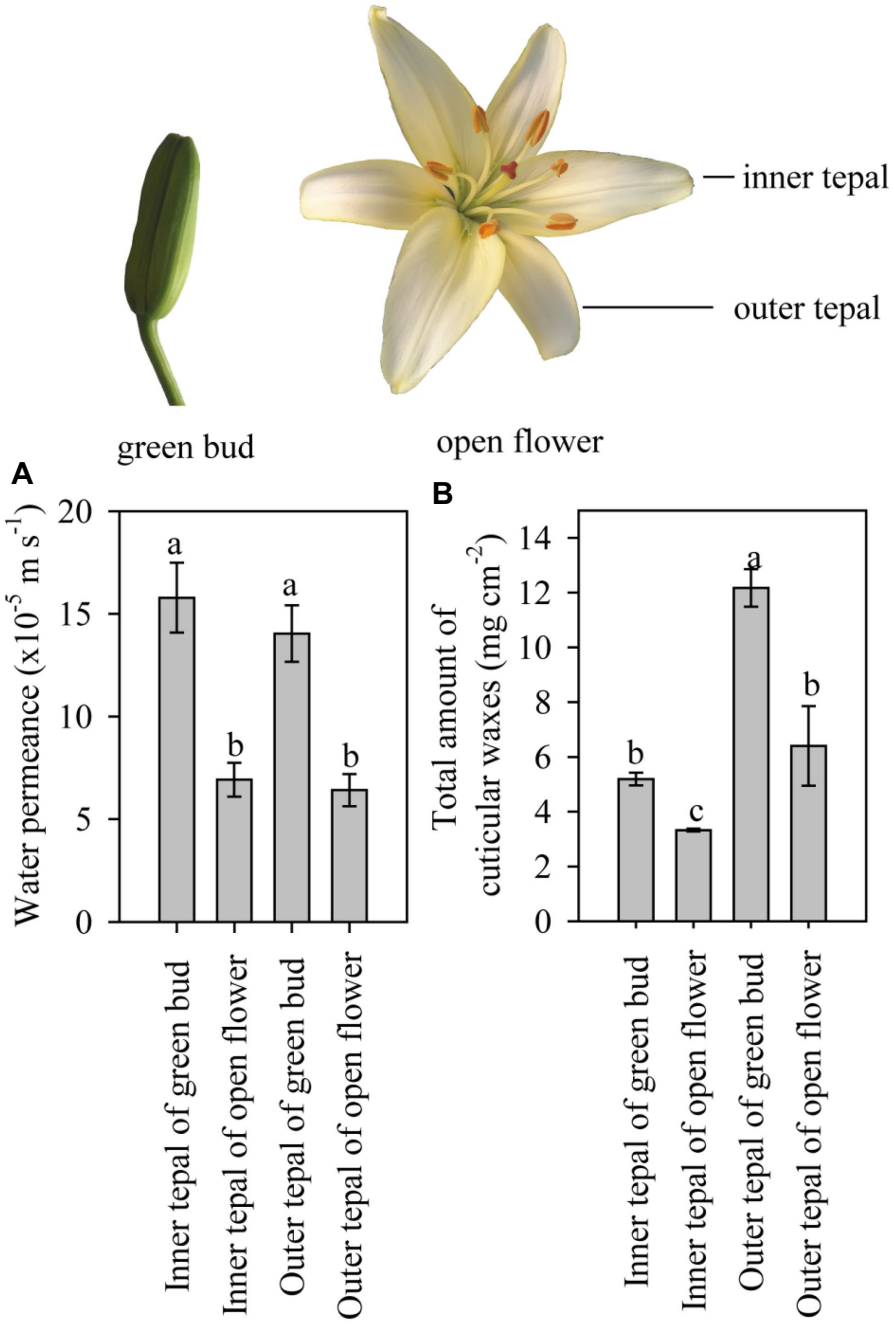
Cuticular transpiration rate and wax coverage of tepals of lily flowers at the green bud and open stages. **(A)** Minimum water conductance and **(B)** total amount of cuticular waxes on the inner and outer tepals of green buds and open flowers of the *Lilium* cultivar ‘Casa Blanca’. Data are presented as the mean±standard deviation (*n*=12 for transpiration, *n*=5 for cuticular wax). The different letters on the bars indicate significant differences (*p*<0.05).

Three *Lilium* spp. cultivars: ‘Casa Blanca’, ‘Huang Tianba’, and ‘Tiber’ were used to evaluate the water permeability and cuticular wax composition differences of the leaf and tepal cuticles. The lily seedlings of all three cultivars were grown in pots filled with soil in a climate chamber at Zhongkai University of Agriculture and Engineering, Guangzhou, China (include coordinates). For the duration of the experiment, the growth conditions were with an average daily light intensity of 100μmolm^−2^ s^−1^ coming from LED lamps (Guangzhou Blueseatec Electronic Co. Ltd., Guangdong, China). The growing was set at a 12/12-h day/night light cycle with a temperature of 20±2°C and a relative humidity of 60±5% in greenhouse. The plants were watered daily to keep the soil moist and facilitate healthy growth. Lily flowers have open tepals and leaves on the stem when harvested (Figure 1). In this study, *Lilium* spp. ‘Casa Blanca’ was selected to comparatively analyse the cuticular transpiration rate and wax composition of the inner and outer tepals at the green bud and open stages (Figure 2), whereas the open tepals and leaves of the *Lilium* spp. ‘Huang Tianba’ and ‘Tiber’ cultivars were used to compare the cuticular transpiration rate and wax composition between different organs. The stem and flower samples for transpiration rate and wax chemical analyses were carefully harvested. Once the experimental plants had reached the appropriate flowering stages, samples were harvested. The flowering stems were cut at 60–80cm in length and immediately placed in water at room ambient approximately 20°C in preparation for analysis.

### Transpiration Rate Determination

The rate of cuticular transpiration from inner and outer tepals of green bud and open flowers of ‘Casa Blanca’ and from the whole tepals and leaves was determined gravimetrically (Cheng et al., 2019). Twelve fresh samples for tepals or leaves from green bud, open flowers or different cultivars without any defects were selected from plants of each cultivar. All samples were fully hydrated prior to evaluation. The petioles of the tepals and leaves were sealed with paraffin wax (melting point approximately 60°C) to avoid water loss from the cut wounds. The temperature (20±2°C) and relative humidity (45±5%) of the surrounding atmosphere were monitored using a digital thermometer (Anymetre, Guangzhou, China). The weight loss of the tepals and leaves was recorded automatically every 30min for 8h using a digital balance with a precision of 0.1mg (BSA-224S; Sartorius, Beijing, China).

The dynamic transpiration changes of leaf and tepal tissues were inflected by the weight loss under different time points. Relative water content (RWC), as an important indicator of water status in plants, was calculated firstly based on full saturation fresh weight (FWs), actual fresh weight (FW) at each measured point, dry weight (DW) and surface area of the measured tissues. RWC was calculated as:


$$RWC=\frac{FW-DW}{FWs-DW}$$

and the RWD was calculated according to the changes of RWC:


RWD=1−RWC

The transpiration rate (flux of water vapor; *F* in g m^−2^ s^−1^) was calculated from the changes in the fresh weight of the samples (*W* in g) over time (Δ*t* in s) and surface area (*A* in m^2^), as follows:


$$F=\frac{\Delta W}{\Delta t\times A}$$

The surface area of the tepals and leaves (*A*) was determined by digital scanning (LaserJet Pro MFP M126nw; HP, Shanghai, China). The water permeance (*P* in m s^−1^) was calculated from the transpiration rate (*F*) divided by the driving force, as follows:


$$P=\frac{F}{c_{WV}^{*}\;\;\left(a_{petal}-a_{air}\right)}$$

where the water vapor saturation concentration at the actual tepal or leaf temperature (water vapor content of air at saturation;) was obtained from tabulated values (Nobel, 2009), the water activity in the air (*a*_air_) was the recorded relative humidity, and the water activity in the tepals or leaves (*a*_tepal_) or (*a*_leaf_), respectively, was assumed to be unity (Burghardt and Riederer, 2003). The minimum water conductance based the drying curve of P *via* RWD was obtained.

### Cuticular Wax Extraction

To extract the surface wax chemicals, tepals of different developmental stages, and from inner and outer positions of ‘Casa Blanca’, inner tepals and leaves of ‘Huang Tianba’ and ‘Tiber’ were harvested. Samples of petals and leaves of intact without defects and cracks were carefully selected, and sample surfaces were genteelly cleaned using a paint brush. Using tweezers to hold the petioles, the tepal and leaf samples were dipped vertically into chloroform (the extract solution). To fully extract the cuticular waxes, each sample was dipped in chloroform three times for 30s each, and the extracts were combined. Then, approximately 5μg *n*-tetracosane was added to the extracts as an internal standard. The solvent was evaporated under a gentle stream of nitrogen and collected for further analysis.

### Wax Composition Analyses

Dried samples were derivatized with *N, O*-bis (trimethylsilyl) trifluoroacetamide in pyridine at 70°C for 30min. To determine the quantity of wax components, samples were analyzed using a capillary gas chromatograph (7820A, GC System; Agilent Technologies, Santa Clara, CA, USA) equipped with a capillary column (30m×0.32mm, DB-1ms, and 0.1-μm-thick film; J&W Scientific, Agilent Technologies). The GC oven was held at 50°C for 2min, raised by 40°C min^−1^ to 200°C, held at 200°C for 2min, and then raised by 3°C min^−1^ to 320°C, and held at 320°C for 30min. The carrier gas was hydrogen. The area of the peaks was compared to that of the internal standard to determine the quantity of wax components.

Wax components were analyzed using a temperature-controlled capillary gas chromatograph equipped with a mass spectrometric detector (*m*/*z* 50–750, MSD 5975; Agilent Technologies) under the same GC conditions as described above but with helium as the carrier gas. Single compounds were identified based on their electron ionization mass spectra using authentic standards, the Wiley 10th/NIST 2014 mass spectral library (W10N14; John Wiley & Sons, 2014), or by interpretation of the spectra according to their retention times, or by comparison with published data.

### Statistical Analysis

Statistical analyses were performed using SPSS (version 23; IBM Corp., Armonk, NY, United States) and SigmaPlot 10 (Systat software, Inc., San Jose, CA, United States). The normal distribution of data was tested *via* a Shapiro–Wilk or Kolmogorov–Smirnov normality test (significance level: *p*<0.05). Comparisons were analyzed using one-way analysis of variance. SigmaPlot 10 was used for graphing.

## Results

### Water Permeance

**Figure 3:**
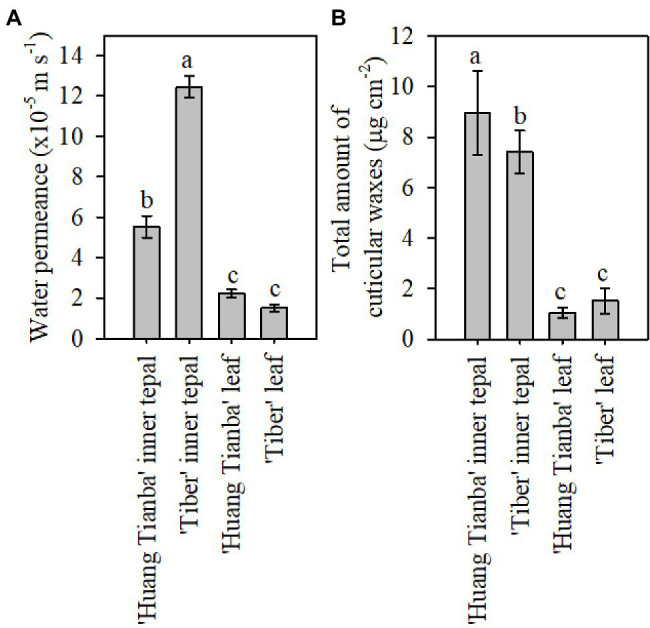
Cuticular transpiration rate and wax coverage of leaves and tepals of lily flowers at the open stage. **(A)** Minimum water conductance, and **(B)** total amount of cuticular waxes on inner tepals and intact leaves of the *Lilium* cultivars ‘Huang Tianba’ and ‘Tiber’. Data are presented as the mean±standard deviation (*n*=12 for transpiration, *n*=5 for cuticular wax). The different lowercase letters on the bars indicate significant differences (*p*<0.05).

Tepals of the ‘Casa Blanca’ cultivar at the green bud stage exhibited higher transpiration rate than tepals at the open stage (Figure 2A), and the mean minimum conductance for the green buds was 1.6×10^−4^ms^−1^ (inner tepals) and 1.4×10^−4^ms^−1^ (outer tepals), whereas a lower minimum water conductance of 6.9×10^−5^ms^−1^ and 6.4×10^−5^ms^−1^ was recorded for inner and outer tepals in open flower, respectively (Figure 2A). Overall, the tepal minimum water conductance was significantly higher compared to that of the leaves (Figure 3A). The water permeance of the inner tepals of the ‘Huang Tianba’ and ‘Tiber’ cultivars was 5.5×10^−5^ms^−1^ and 1.3×10^−4^ms^−1^, respectively. In comparison with tepals, the minimum water conductance of the leaves was much lower, being 2.2×10^−5^ms^−1^ for the ‘Huang tianba’ cultivar and 1.5×10^−5^ms^−1^ for the ‘Tiber’ cultivar (Figure 3A).

### Wax Load and Chemical Composition at Different Tepal Development Stages

As the primary barrier to non-stomatal transpiration is determined by the wax pattern in the cuticle (Riederer and Schreiber, 2001), the composition of the wax on the leaves and tepals at the green bud and open flower stages was analyzed. The overall wax coverage was 12.17±0.69μgcm^−2^ and 6.40±1.45μgcm^−2^ on the outer tepals of the green bud and open flowers, respectively (Figure 2B). Lower amounts of wax were detected on the inner tepals of the green buds and open flowers at 5.19±0.23μgcm^−2^ and 3.33±0.05μgcm^−2^, respectively (Figure 2B).

**Figure 4:**
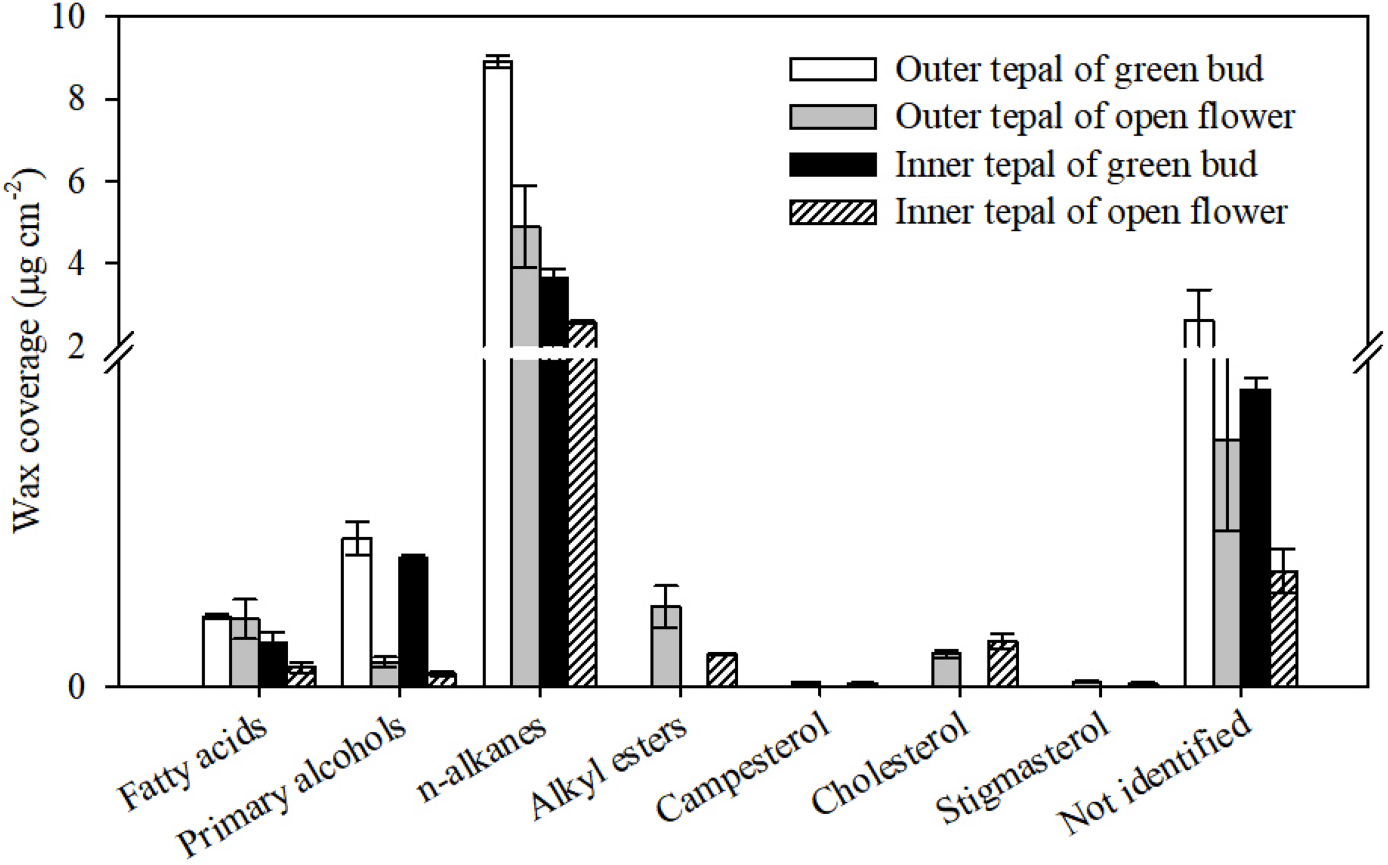
Chemical composition of cuticular waxes on green bud and open flower tepals of *Lilium* cultivar ‘Casa Blanca’. Data are presented as the mean±standard deviation (*n*=5).

The waxes on the tepals of the green buds and open flowers were composed of very-long-chain fatty acids, primary alcohols, and *n*-alkanes (Figure 4). The most abundant wax components were *n*-alkanes, which comprised 73.28% (of the total wax) on the outer tepals and 70.27% on the inner tepals at the green bud stage, and 76.68% on the outer tepals and 77.34% on the inner tepals of the open flowers (Figure 4). Primary alcohols occurred also in relatively higher proportions in the cuticle on the green bud tepals (4.09% outer and 8.30% inner) compared to that on the tepals of the open flowers (1.29% outer and 6.07% inner, Figure 4). There were no obvious differences in the fatty acid content of the wax on the tepals at the green bud and open flower stages. Very-long-chain alkyl esters and cyclic sterols were detected only in the wax on the tepals of the open flowers (Figure 4; Supplementary Table S1).

**Figure 5:**
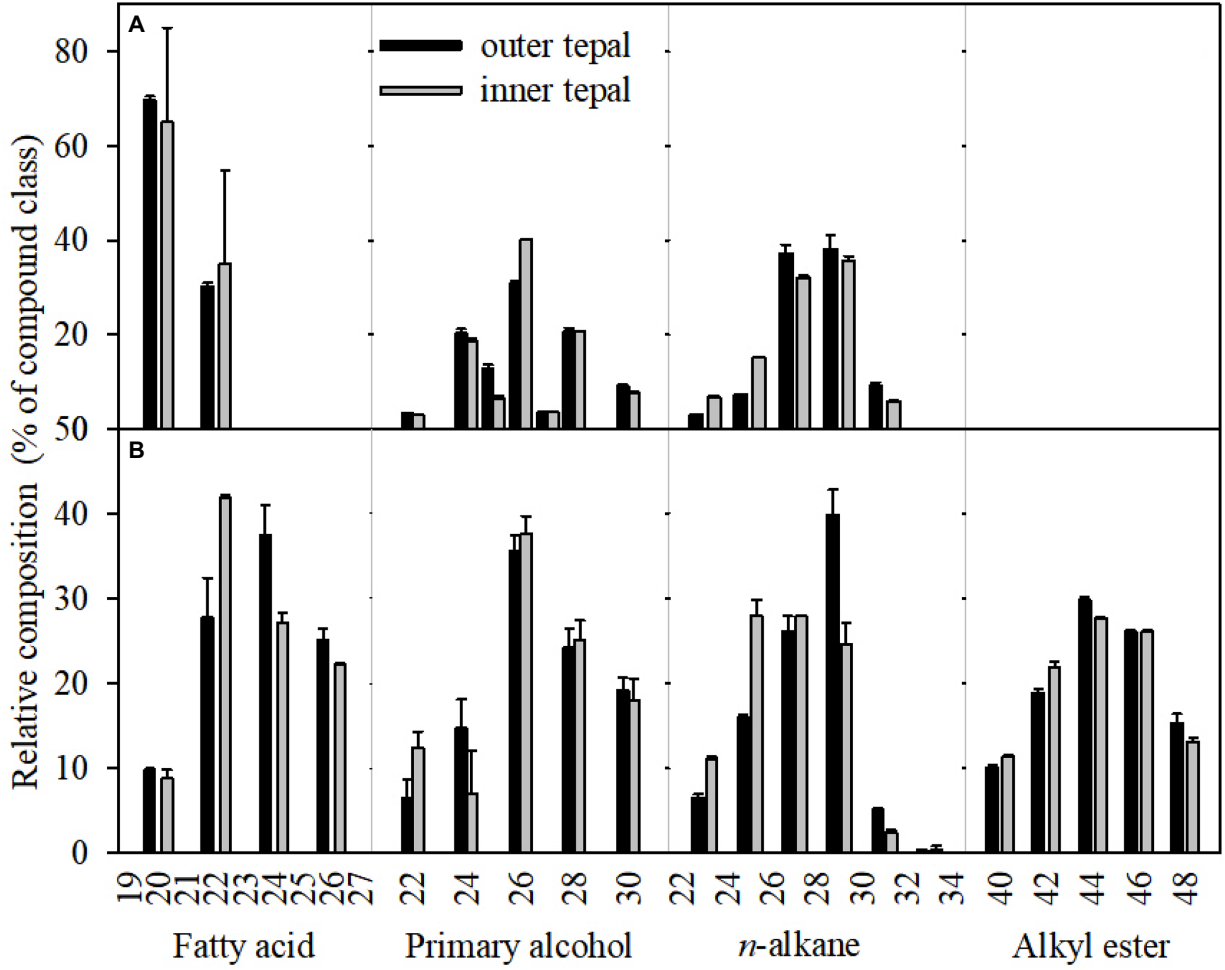
Carbon chain-length distribution of the cuticular waxes on the tepals of green buds **(A)** and open flowers **(B)** of the *Lilium* cultivar ‘Casa Blanca’. Data are presented as the mean±standard deviation (*n*=5).

Acyclic components dominated the wax mixtures on the tepals in green buds and open flowers. The prominent components of the *n*-alkanes had chain lengths ranged from C_22_ to C_33_, with most abundant of C_27_ and C_29_. The C_33_ *n*-alkane was detected only in the cuticle of tepals at the open flower stage (Figure 5; Supplementary Table S1). Chain length of primary alcohols ranged from C_22_ to C_30_, with a dominant chain length of C_26_. In addition, primary alcohols with chain lengths of C_28_ and C_30_ were more abundant in outer and inner tepals at the open flower stage (24.21 and 19.10%, respectively) compared to that of green bud tepals (25.04 and 18.00%, respectively). The fatty acid fraction in the green bud cuticles were dominated by C_20_ and C_22_, whereas chain lengths ranged from C_20_ to C_26_ for fatty acid in the open flower cuticles. The alkyl esters, which were detected only in the cuticles of the open flower tepals, possessed chain lengths ranging from C_40_ to C_48_ (Figure 5; Supplementary Table S1).

### Cuticular Waxes of Open Flower Tepals and Leaves in Different Cultivars

Comparative profiling of the cuticular waxes on the tepals and leaves of the ‘Huang Tianba’ and ‘Tiber’ cultivars was conducted. The overall amount of cuticular wax on the inner tepals was 8.95±1.68μgcm^−2^ for the ‘Huang Tianba’ cultivar, which was higher than that for the inner tepals of the ‘Tiber’ cultivar (7.41±0.85μgcm^−2^; Figure 3B). Compared to the significant difference in wax quantity on the tepals of the two cultivars, the amount of wax on the leaves showed only a slight variation between cultivars at 1.04±0.20μgcm^−2^ for ‘Huang Tianba’, and 1.52±0.51μgcm^−2^ for ‘Tiber’ (Figure 3B).

**Figure 6:**
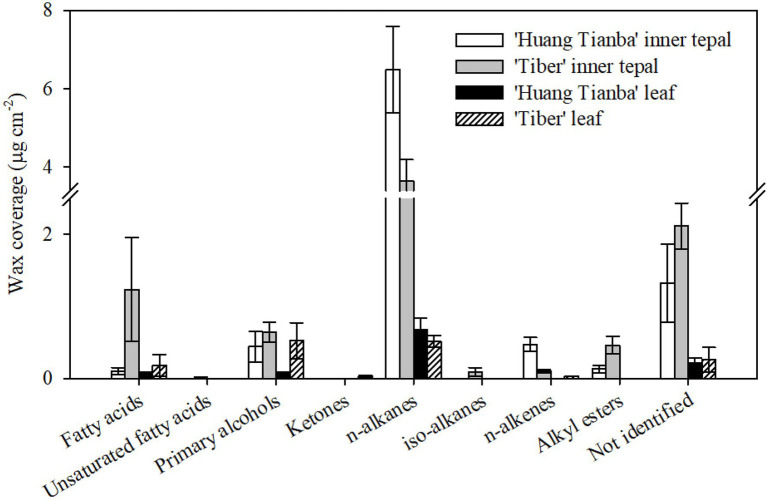
Chemical composition of cuticular waxes on the tepals and leaves of the *Lilium* cultivars ‘Huang Tianba’ and ‘Tiber’. Data are presented as the mean±standard deviation (*n*=5).

The wax mixtures of both tepal and leaf cuticles were dominated by very-long-chain acyclic components such as fatty acids, primary alcohols, *n*-alkanes, alkyl esters, and small amounts of branched *n*-alkanes and *n*-alkenes (Figure 6; Supplementary Table S2). The most abundant aliphatic fraction in both the tepal and leaf waxes comprised *n*-alkanes, which contributed 72.49 and 65.27% for the ‘Huang Tianba’ cultivar, and 44.09 and 33.03% for the ‘Tiber’ cultivar, respectively. The coverage of *n*-alkanes was significantly higher on the surface of the tepals compared to that on the leaf surfaces for both cultivars (Figure 6; Supplementary Table S2). The primary alcohols contributed 4.93 and 7.07% (‘Huang Tianba’), and 7.68 and 33.99% (‘Tiber’) of the tepal and leaf wax coverage, respectively. Compared to the relatively small concentrations of fatty acids in the tepal and leaf cuticles of the ‘Huang Tianba’ cultivar (1.16 and 7.31%, respectively), higher fatty acid levels were found in the ‘Tiber’ tepal and leaf cuticles (14.84 and 11.72%, respectively, Figure 6; Supplementary Table S2). Small amounts of *n*-alkenes and branched *n*-alkanes were detected in the tepal cuticles of both cultivars, and traces were found in the leaf cuticle of the ‘Tiber’ cultivar. Alkyl esters were not detected in the leaf waxes, but occurred in the tepal waxes of both cultivars. In addition, traces or no signals of cyclic compounds were detected in the tepal and leaf waxes of both the ‘Huang Tianba’ and ‘Tiber’ cultivars (Figure 6; Supplementary Table S2).

**Figure 7:**
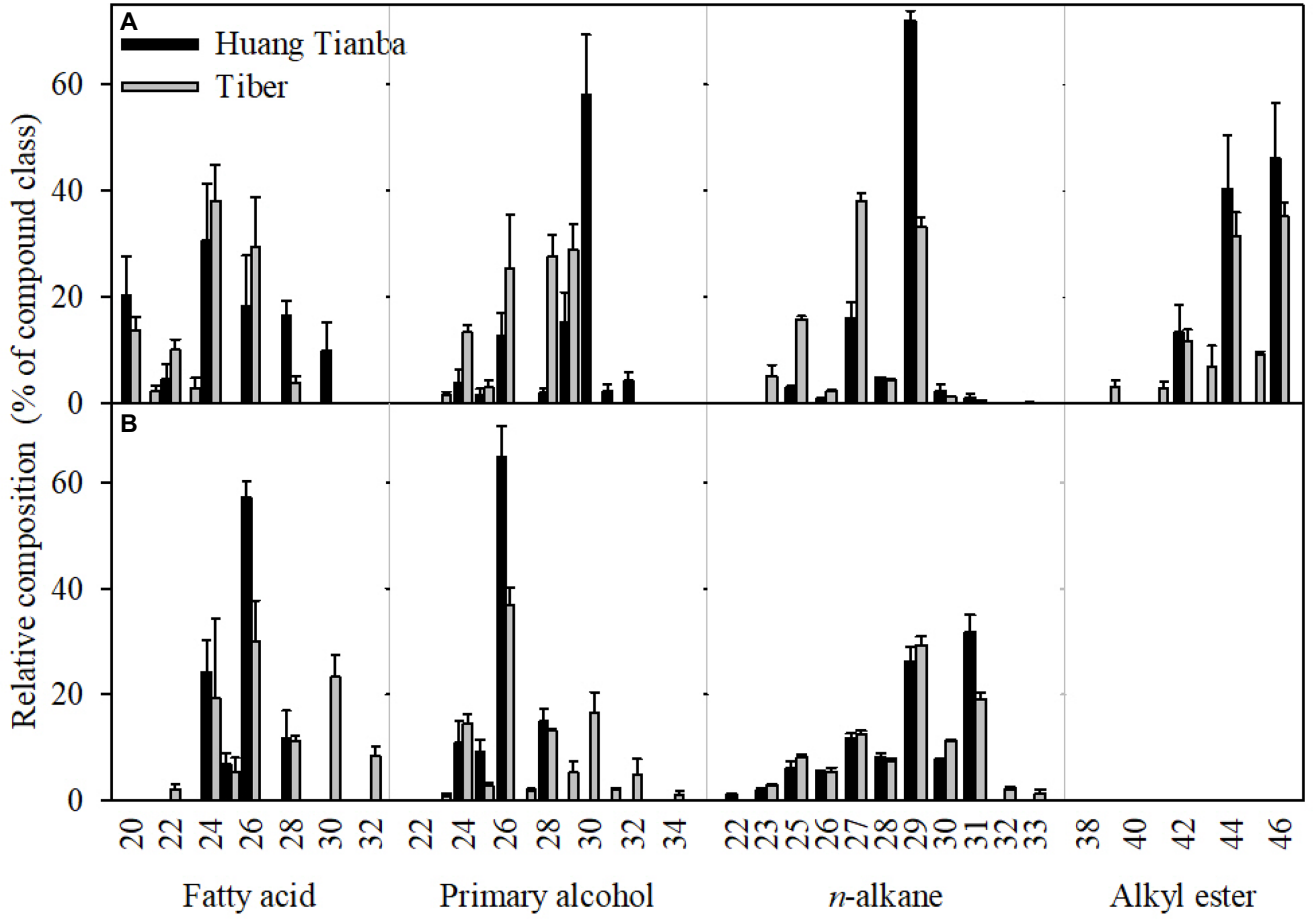
Carbon chain-length distribution of the cuticular waxes on the inner tepals **(A)** and leaves **(B)** of the *Lilium* cultivars ‘Huang Tianba’ and ‘Tiber’. Data are presented as the mean±standard deviation (*n*=5).

The chain-length distributions were similar among the compound classes within cultivars, whereas the predominant carbon chain lengths were substantially different between organs. The chain lengths of the *n*-alkanes, which were predominantly odd-number carbon chains, ranged from C_22_ to C_33_, with mostly C_29_ (‘Huang Tianba’) and C_27_ (‘Tiber’) in the tepal cuticles, and C_31_ (‘Huang Tianba’) and C_29_ (‘Tiber’) chain lengths in the leaf waxes (Figure 7; Supplementary Table S2).

A broad range of primary alcohol chain lengths (C_23_ to C_34_) was found, with prominent of C_30_ and C_26_ in the tepal cuticles and leaf cuticles of both cultivars. In both cultivars, the chain lengths of the fatty acid fraction ranged from C_20_ to C_30_, with the C_24_ chain length predominantly in the tepal cuticles. However, leaf cuticles contained fatty acids with chain lengths ranging from C_20_ to C_32_, with a predominance of the C_26_. The unbranched alkyl esters, which were detected only in the tepal waxes, ranged in chain length from C_39_ to C_46_ (Figure 6). In addition, *n*-alkenes ranged in chain length from C_25_ to C_31_, with a dominant chain length of C_29_ in the tepal waxes. Ketones with chain lengths of C_29_ and C_31_ were detected in the leaf cuticles of the ‘Tiber’ cultivar (Figure 7; Supplementary Table S2).

## Discussion

The transpiration of cut flowers has been recently cared by researchers on different species and flower organs. As the occurrence of stomata, water loss occurred mainly through the leaf tissues in the intact cut flowers. When most of the stomata closed, leaves were still the main parts for water loss in roses, but not in carnation or chrysanthemum (Lin et al., 2020; Tsaniklidis et al., 2020; Fanourakis et al., 2021). These studies considered stomatal transpiration as the main pathway for water loss in cut flowers. However, the non-functional or non-stomatal transpiration, which has been widely reported to be the cuticular transpiration for leaves and fruit (Riederer and Schreiber, 2001), whereas relatively little is available for flowers. So far, only two studies reported the cuticular transpiration of flowers. The water permeability of intact petal of *C. bipinnatus* “Sensation” was 6.7×10^−5^ms^−1^ (Buschhaus et al., 2015). Our previous study on rose petals found similar cuticular transpiration levels with 4.1×10^−5^ms^−1^ for the ‘Movie star’ and 8.9×10^−5^ms^−1^ for ‘Tineke’ (Cheng et al., 2019). Cut lily flowers contains flowering head with tepals, leaves, and stem (Figure 1; van Doorn and Han, 2011). Stomata occurred on the abaixal side of leaf and outer tepal surfaces of lily flowers (Supplementary Figure S1). The minimum conductance was used to indicate the cuticular transpiration or with non-functional stomata transpiration (Burghardt and Riederer, 2003). Leaf of lily flowers exhibited typical drying curves as reported previously (Supplementary Figure S2). The water loss of petals in lily flowers exhibited almost linearly following the time changes during the whole dehydration process (Supplementary Figures S3, S4). In addition, the stomata on lily tepals were shown covered by waxes or under closed status (Supplementary Figure S1). Therefore, the drying curve and water loss dynamic changes implied that the stomata of sepals might be non-functional.

The mean minimum conductance of the wax layer on the tepals of the green bud stage of the lily cultivar was higher than the tepals of the open flowers, which showed similar levels as reported results (Cheng et al., 2019). In a number of studies on leaf tissue transpiration, the reported water permeance ranges from 2.6×10^−7^ms^−1^ for *Zamioculcas zamiifolia* (G.Lodd.) Engl. to 4×10^−3^ms^−1^ for *Ipomoea batatas* (L.) Lam., with most values lower than 2×10^−4^ms^−1^ (Schuster et al., 2017). The minimum water conductance of the cuticles on the green bud and open flower tepals and on the leaves of all three lily cultivars showed a similar level to that of most of the reported values. In addition, the minimum water conductance exhibited by the lily cuticles was higher in tepals than in leaves (Figure 3A). Taking water loss of petal and leaf cuticles in *C. bipinnatus* and rose cultivars (Buschhaus et al., 2015; Cheng et al., 2019) together, the wax layer on tepals and petals appears to be a less efficient barrier to transpiration compared to that on leaves. This might be related to the special functions of petals, which may have rapid cuticular transpiration together with volatilization of aroma to attract pollinators. On the other hand, flowers are relatively short-lived organs even when environmental conditions are favorable. Thus, it is probably not necessary for flowers to develop such a highly efficient transpiration barrier, compared to that needed on long-lived leaves. The cuticular wax loading on petals has been reported for only a few species, with values ranging from 2.7μgcm^−2^ (*C. bipinnatus*; Buschhaus et al., 2015) to 37μgcm^−2^ (*T. officinale*; Guo and Jetter, 2017). Only one study (on *Antirrhinum majus* L., the snapdragon) has examined the cuticular wax loading during petal development (Goodwin et al., 2003). By contrast, the accumulation of waxes on the tepals decreased substantially from the green bud to the open flower stage, for both the inner and outer tepals of the *Lilium* cultivar ‘Casa Blanca’. The overall tepal wax coverage was not significantly different between the cultivars ‘Huang Tianba’ and ‘Tiber’. However, wax coverage was higher on the tepals than on the leaves. As reported previously, wax coverage on the lily tepals exhibited cultivar-specific differences (Huang et al., 2017; Cheng et al., 2019). In addition, water permeance of the lily leaves was significantly lower than that of the tepals, supporting the assertion that there is no significant relationship between the cuticular water permeability and the total amount of wax (Riederer and Schreiber, 2001; Jetter and Riederer, 2016).

The chemical composition of petal cuticles differs among different flowers. The wax layers on the petals of *Solanum tuberosum* L. (potato) and *A. majus* (snapdragon) flowers contained relatively high concentrations of *n*-alkanes and methyl-branched alkanes (Goodwin et al., 2003; Guo and Jetter, 2017). Similarly, the major cuticular wax components of *Vicia faba* L. (faba bean) and *Rubus idaeus* L. (raspberry) petals were *n*-alkanes (Griffiths et al., 1999, 2000). Primary alcohols (21%) and *n*-alkanes (29%) were found in similar amounts in the wax layer of *T. officinale* (Guo et al., 2017). Our current study found that the waxes on petals of the rose cultivars ‘Movie star’ and ‘Tineke’ were dominated by *n-*alkanes (46.8 and 64.3%) and *n*-alkenes (47.3 and 20.2%; Cheng et al., 2019). Similarly, in the present study, *n*-alkanes were found to be the major components of the lily tepal waxes (Figures 4, 6). However, primary alcohols and alkyl esters were the predominant components in petal waxes of *C. bipinnatus* and *Petunia hybrida* Vilm. W115 (Petunia) flowers, whereas *n*-alkanes and triterpenoids occurred at low levels (King et al., 2007; Buschhaus et al., 2015). Minor amounts of branched *n*-alkanes or *n*-alkenes were found in the tepal waxes of the lily cultivars ‘Huang Tianba’ and ‘Tiber’, and sterols were found in the tepal waxes of open lily flowers (Figures 4, 6).

It has been proposed that the hydrophobic barrier of the cuticle is mainly determined by the chemical composition and chain-length distribution of the very-long-chain aliphatics and their structural arrangements (Riederer and Schreiber, 1995). The accumulation of very-long-chain components in the wax layer shift from C_16_ and C_18_ to longer than C_20_ carbon chains during the development of leaves in *Arabidopsis* (Jenks et al., 1995; Busta et al., 2017) and *Prunus laurocerasus* (Jetter and Schäffer, 2001). In maize, the predominant aliphatic components in the leaf cuticle were C_21,_ C_23_, and C_25_ *n*-alkanes at early stages of development but shifted to chain lengths over C_28_ in later development (Bourgault et al., 2020). Similarly, in the present study, the fatty acid fraction in the tepal waxes of the green bud stage mainly contained C_20_ and C_22_, whereas they ranged from C_20_ to C_26_ in the open flower tepal cuticles (Figure 5; Supplementary Tables S1 and S2). The *n*-alkanes with chain lengths over C_31_ and alkyl esters over C_36_ were detected only in the cuticles on the tepals of open flowers (Figure 5). A shift to longer hydrocarbon chain lengths is due to the bio-synthesis of waxes regulated by a series of genes and enzymes, as well as their functional properties during developmental stages (Busta et al., 2017).

Previous studies indicated that C-chains of cuticular waxes accumulating in petal waxes are shorter compared to those in leaf waxes. *C. bipinnatus* petal and leaf waxes are dominated by primary alcohols and fatty acids with carbon chain lengths of C_22_ and C_24_, and C_28_ and C_30_, respectively (Buschhaus et al., 2015). The waxes on the flowers and leaves of *T. officinale* contained branched C_27_ to C_31_, and non-branched C_27_ to C_33_ *n*-alkanes (Guo and Jetter, 2017). Alcohol esters with a predominance of C_22_ to C_28_, and C_28_ to C_32_ were found in *P. hybrida* flower and leaf waxes, respectively (King et al., 2007). In *Arabidopsis*, *n*-alkanes with a chain length of C_29_ dominate the aliphatic pattern in petal waxes, whereas a chain length of C_31_ is dominant in the leaf waxes (Jenks et al., 1995; Shi et al., 2011). In rose cultivars ‘Movie star’ and ‘Tineke’, the predominant chain lengths are C_31_ and C_33_ *n*-alkanes in the leaf waxes, whereas C_27_ and C_29_ *n*-alkanes and *n*-alkenes dominate the petal waxes (Cheng et al., 2019). In the present study, the chain lengths of the *n*-alkanes ranged from C_22_ to C_33_, with C_27_ and C_29_ chain lengths predominating in the tepal waxes, whereas chain lengths of C_31_ and C_29_ were dominant in the leaf waxes of the ‘Huang Tianba’ and ‘Tiber’ cultivars, respectively (Figure 7). These findings corroborated the typical chain-length distributions of the aliphatic component, being shorter in petal waxes compared to that in leaf waxes (Buschhaus et al., 2015; Jetter and Riederer, 2016).

In contrast to the chemical analysis of petal waxes, few studies have investigated the transpiration barrier properties of petal cuticles. A study on water permeability of petal waxes has been conducted for *C. bipinnatus* (Buschhaus et al., 2015). As mentioned above, the relatively high concentrations of C_22_ and C_24_ carbon chain components in *C. bipinnatus* petal waxes are thought to create a less efficient barrier to transpiration compared to that of the leaf cuticles. Similar results have been found for rose petal waxes in the ‘Movie star’ and ‘Tineke’ cultivars, which accumulated mainly C_26_ and C_29_, resulting in higher cuticular transpiration compared to that in leaves with a predominance of C_31_ (Cheng et al., 2019). Patwari et al. (2019) found that the lack of very-long-chain wax esters induced a higher cuticular permeability in *wsd1* mutants in *Arabidopsis*. Taken previous results together with present study on lily, C-chain lengths distribution in cuticular wax might play a pivotal role in limiting non-stomatal transpiration in plant tissues. Flowers that accumulate very long chain of aliphatics might exhibit slower weight loss and longer shelf life during marketing.

In conclusion, cuticular transpiration of lily flower green bud tepals was significantly higher than that of the open flower tepals and was significantly higher in the tepals than in the leaves. The most abundant aliphatic compounds in the tepal waxes of the lily cultivars ‘Casa Blanca’ and ‘Huang Tianba’ were *n*-alkanes, whereas large amounts of primary alcohols were detected in the tepal waxes of the ‘Tiber’ cultivar. In addition, there was a predominance of relatively short carbon chain aliphatics in the tepal waxes of the green bud stage, compared to the chain lengths in tepal waxes of the open flower stage, and similar differences between the tepal and leaf waxes were found. These findings suggest that the less efficient transpiration barrier properties of the tepal waxes are probably related to the accumulation of relatively short-chain aliphatics. Furthermore, cuticular transpiration and the barrier properties of the cuticular waxes of lily cultivars exhibited organ- and cultivar-specific differences. These findings provide further insight to link the cuticular chemicals with functional properties as well as with the physiological adaptations of plant tissues. The findings of this study also contribute to the cultivar selection for flowers, in which accumulation of very long chain of aliphatics might exhibit slower weight loss and longer shelf life during marketing. The cuticular barrier properties are varied in organ-, cultivar-, and species differences, which are largely affected by the accumulation of chemical compositions and arrangement of cuticle (Riederer and Schreiber, 2001; Huang et al., 2017). In addition, the growing conditions have also been widely reported to be one of the most important factors in influencing the transpiration changes. Therefore, selecting appropriate cultivar, setting appropriate growing conditions including water and light availability, temperature and humidity control, etc., will largely influence the water loss of flowers. Combining post-harvest treatments such as nano-silver solution with sugars to spray on flowers forms thin aqueous membrane, slowing down the rapid post-harvest changes (Naing and Kim, 2020). Therefore, caring the transpiration barrier properties will be helpful for the development of techniques with the potential to extend the shelf life of cut flowers based on the transpiration barrier properties of the cuticular waxes after harvest.

## Data Availability Statement

The original contributions presented in the study are included in the article/Supplementary Material, further inquiries can be directed to the corresponding author.

## Author Contributions

HH and GC designed the project and prepared the manuscript. GC and LW performed most of the experiments. HW and XY took part in part of the experiments. NZ, XW, and LH helped to make interpretation of the results. All authors contributed to the article and approved the submitted version.

## Funding

This work was supported by the Characteristic Innovation of Project of Education Department of Guangdong Province (Natural Science 2020KTSCX053), the Common Technical Innovation Team of Guangdong Province on Preservation and Logistics of Agricultural Products (2019KJ145), the Foundation of Guangxi Key Laboratory of Fruits and Vegetables Storage-Processing Technology (2021-01), and the special fund for scientific innovation strategy-construction of high level Academy of Agriculture Science of Guangdong Academy of Agricultural Sciences (R2019QD-012).

## Conflict of Interest

The authors declare that the research was conducted in the absence of any commercial or financial relationships that could be construed as a potential conflict of interest.

## Publisher’s Note

All claims expressed in this article are solely those of the authors and do not necessarily represent those of their affiliated organizations, or those of the publisher, the editors and the reviewers. Any product that may be evaluated in this article, or claim that may be made by its manufacturer, is not guaranteed or endorsed by the publisher.
