# Supplementary material for: Variation in Petal and Leaf Wax Deposition Affects Cuticular Transpiration in Cut Lily Flowers
Source: Front Plant Sci. 2021 Nov 24;12:781987. doi: 10.3389/fpls.2021.781987 (PMC8652255; doi:10.3389/fpls.2021.781987)
Supplement: Supplementary file 1 [file Data_Sheet_1.docx]

**Variation in petal and leaf wax deposition affects cuticular transpiration in cut lily flowers**

Guiping Cheng^1^, Lin Wang^3^, Hairong Wu^4^, Xinfan Yu^1^, Nan Zhang^2^, Xiaorong Wan^1^, Lihong He^1^, Hua Huang^2,*^

*^1^College of Agriculture and Biology,* *Zhongkai University of Agriculture and Engineering, Guangzhou 510225, P.R. China*

*^2^Institute of Fruit Tree Research, Guangdong Academy of Agricultural Sciences; Key Laboratory of South Subtropical Fruit Biology and Genetic Resource Utilization*, *Ministry of Agriculture and Rural Affairs,Guangdong Provincial Key Laboratory of Tropical and Subtropical Fruit Tree Research, Guangzhou, 510640,  P. R. China*

*^3^Sericultural & Agri-Food Research Institute Guangdong Academy of Agricultural Sciences, Key Laboratory of Functional Foods, Ministry of Agriculture and Rural Affairs, Key Laboratory of Agricultural Products Processing, Guangzhou 510610, P. R. China*

*^4^Customs Technology Center of Guangzhou Customs District, Guangzhou 510623, P.R. China*

* **Corresponding Author**

Dr. Huang Hua

1. mail: [huangw0109@gmail.com](mailto:huangw0109@gmail.com)

**Supplementary table S1** Detailed wax composition of *Lilium* var ‘Casa blanca’ tepals (*µ*g cm^-2^). Data were given as means ± standard deviations (*n* = 5). Value lower than 0.01*µ*g cm^-2^ is recorded as trace.

|  |  | Outside tepal of green bud | | |  | Outside tepal of open flower | | |  | Inside tepal of green bud | | |  | Inside tepal of open flower | | |
| --- | --- | --- | --- | --- | --- | --- | --- | --- | --- | --- | --- | --- | --- | --- | --- | --- |
| Fatty acid | 20 | 0.112 | ± | 0.004 |  | 0.018 | ± | 0.005 |  | 0.078 | ± | 0.040 |  | 0.004 | ± | 0.003 |
|  | 22 | 0.048 | ± | 0.000 |  | 0.051 | ± | 0.007 |  | 0.036 | ± | 0.006 |  | 0.016 | ± | 0.004 |
|  | 24 |  |  |  |  | 0.072 | ± | 0.028 |  |  |  |  |  | 0.011 | ± | 0.005 |
|  |  |  |  |  |  |  |  |  |  |  |  |  |  |  |  |  |
| Primary alcohol | 22 | 0.018 | ± | 0.001 |  | 0.005 | ± | 0.001 |  | 0.013 | ± | 0.001 |  | 0.005 | ± | 0.002 |
|  | 24 | 0.111 | ± | 0.001 |  | 0.012 | ± | 0.000 |  | 0.080 | ± | 0.002 |  | 0.003 | ± | 0.003 |
|  | 25 | 0.070 | ± | 0.007 |  |  |  |  |  | 0.028 | ± | 0.002 |  |  |  |  |
|  | 26 | 0.168 | ± | 0.002 |  | 0.030 | ± | 0.007 |  | 0.173 | ± | 0.007 |  | 0.016 | ± | 0.001 |
|  | 27 | 0.017 | ± | 0.001 |  |  |  |  |  | 0.016 | ± | 0.000 |  |  |  |  |
|  | 28 | 0.113 | ± | 0.007 |  | 0.020 | ± | 0.006 |  | 0.089 | ± | 0.003 |  | 0.011 | ± | 0.001 |
|  | 30 | 0.050 | ± | 0.002 |  | 0.016 | ± | 0.004 |  | 0.033 | ± | 0.002 |  | 0.008 | ± | 0.001 |
| *n*-alkane | 22 |  |  |  |  | 0.011 | ± | 0.001 |  |  |  |  |  | 0.009 | ± | 0.001 |
|  | 23 | 0.263 | ± | 0.004 |  | 0.316 | ± | 0.044 |  | 0.246 | ± | 0.017 |  | 0.286 | ± | 0.019 |
|  | 25 | 0.649 | ± | 0.004 |  | 0.780 | ± | 0.143 |  | 0.549 | ± | 0.037 |  | 0.719 | ± | 0.030 |
|  | 26 |  |  |  |  | 0.094 | ± | 0.014 |  |  |  |  |  | 0.059 | ± | 0.005 |
|  | 27 | 3.313 | ± | 0.155 |  | 1.273 | ± | 0.176 |  | 1.171 | ± | 0.074 |  | 0.718 | ± | 0.006 |
|  | 28 | 0.483 | ± | 0.043 |  | 0.152 | ± | 0.019 |  | 0.171 | ± | 0.025 |  | 0.069 | ± | 0.005 |
|  | 29 | 3.386 | ± | 0.273 |  | 1.967 | ± | 0.539 |  | 1.302 | ± | 0.081 |  | 0.632 | ± | 0.050 |
|  | 30 |  |  |  |  | 0.037 | ± | 0.009 |  |  |  |  |  | 0.010 | ± | 0.006 |
|  | 31 | 0.811 | ± | 0.060 |  | 0.251 | ± | 0.043 |  | 0.212 | ± | 0.018 |  | 0.062 | ± | 0.008 |
|  | 33 |  |  |  |  | 0.015 | ± | 0.003 |  |  |  |  |  | 0.009 | ± | 0.009 |
| Alkyl ester | 40 |  |  |  |  | 0.027 | ± | 0.006 |  |  |  |  |  | 0.012 | ± | 0.001 |
|  | 42 |  |  |  |  | 0.050 | ± | 0.012 |  |  |  |  |  | 0.024 | ± | 0.001 |
|  | 44 |  |  |  |  | 0.080 | ± | 0.020 |  |  |  |  |  | 0.030 | ± | 0.001 |
|  | 46 |  |  |  |  | 0.070 | ± | 0.019 |  |  |  |  |  | 0.028 | ± | 0.001 |
|  | 48 |  |  |  |  | 0.041 | ± | 0.014 |  |  |  |  |  | 0.014 | ± | 0.001 |
| campesterol |  |  |  |  |  | 0.014 | ± | 0.002 |  |  |  |  |  | 0.012 | ± | 0.002 |
| cholesterol |  |  |  |  |  | 0.110 | ± | 0.013 |  |  |  |  |  | 0.152 | ± | 0.026 |
| stigmasterol |  |  |  |  |  | 0.017 | ± | 0.002 |  |  |  |  |  | 0.009 | ± | 0.006 |
|  |  |  |  |  |  |  |  |  |  |  |  |  |  |  |  |  |
| unkown |  | 2.560 | ± | 0.677 |  | 0.827 | ± | 0.303 |  | 0.997 | ± | 0.040 |  | 0.387 | ± | 0.074 |
| sum of acyclic components |  | 9.561 | ± | 0.064 |  | 5.435 | ± | 1.134 |  | 4.197 | ± | 0.245 |  | 2.767 | ± | 0.009 |
| sum of cyclic components |  |  |  |  |  | 0.141 | ± | 0.017 |  |  |  |  |  | 0.173 | ± | 0.024 |
| Total |  | 12.172 | ± | 0.685 |  | 6.403 | ± | 1.454 |  | 5.194 | ± | 0.232 |  | 3.327 | ± | 0.049 |

**Supplementary table S2** Detailed wax composition of *Lilium* var ‘Huang tianba’ and ‘Tiber’ inside tepal and leaf (*µ*g cm^-2^). Data were given as means ± standard deviations (*n* = 5). Value lower than 0.01*µ*g cm^-2^ is recorded as trace.

|  |  | ‘Huang tianba' inside tepal | | |  | ‘Tiber' inside tepal | | |  | ‘Huang tianba' leaf | | |  | ‘Tiber' leaf | | |
| --- | --- | --- | --- | --- | --- | --- | --- | --- | --- | --- | --- | --- | --- | --- | --- | --- |
| Fatty acids (Saturated) | 20 | 0.02 | ± | 0.03 |  | 0.05 | ± | 0.02 |  |  |  |  |  |  |  |  |
|  | 21 |  |  |  |  | 0.01 | ± | 0.00 |  |  |  |  |  |  |  |  |
|  | 22 | trace |  |  |  | 0.03 | ± | 0.01 |  |  |  |  |  | trace |  |  |
|  | 23 |  |  |  |  | 0.01 | ± | 0.01 |  |  |  |  |  |  |  |  |
|  | 24 | 0.03 | ± | 0.04 |  | 0.13 | ± | 0.05 |  | 0.02 | ± | 0.00 |  | 0.04 | ± | 0.04 |
|  | 25 |  |  |  |  |  |  |  |  | 0.01 | ± | 0.00 |  | 0.01 | ± | 0.00 |
|  | 26 | 0.02 | ± | 0.02 |  | 0.10 | ± | 0.02 |  | 0.04 | ± | 0.00 |  | 0.04 | ± | 0.03 |
|  | 28 | 0.02 | ± | 0.02 |  | 0.01 | ± | 0.01 |  | 0.01 | ± | 0.00 |  | 0.02 | ± | 0.01 |
|  | 30 | 0.01 | ± | 0.01 |  |  |  |  |  |  |  |  |  | 0.04 | ± | 0.04 |
|  | 32 |  |  |  |  |  |  |  |  |  |  |  |  | 0.01 | ± | 0.01 |
|  |  |  |  |  |  |  |  |  |  |  |  |  |  |  |  |  |
| Unsaturated fatty acid | 20 |  |  |  |  | 0.01 | ± | 0.01 |  |  |  |  |  |  |  |  |
|  |  |  |  |  |  |  |  |  |  |  |  |  |  |  |  |  |
| Primary alcohols | 23 |  |  |  |  | 0.01 | ± | 0.00 |  |  |  |  |  | trace |  |  |
|  | 24 | 0.01 | ± | 0.02 |  | 0.09 | ± | 0.02 |  | 0.01 | ± | 0.00 |  | 0.07 | ± | 0.03 |
|  | 25 | 0.01 | ± | 0.01 |  | 0.02 | ± | 0.01 |  | 0.01 | ± | 0.00 |  | 0.01 | ± | 0.00 |
|  | 26 | 0.06 | ± | 0.06 |  | 0.16 | ± | 0.05 |  | 0.05 | ± | 0.01 |  | 0.19 | ± | 0.07 |
|  | 27 |  |  |  |  |  |  |  |  |  |  |  |  | 0.01 | ± | 0.00 |
|  | 28 | 0.01 | ± | 0.01 |  | 0.18 | ± | 0.05 |  | 0.01 | ± | 0.00 |  | 0.07 | ± | 0.04 |
|  | 29 | 0.06 | ± | 0.06 |  | 0.19 | ± | 0.06 |  |  |  |  |  | 0.03 | ± | 0.01 |
|  | 30 | 0.27 | ± | 0.27 |  |  |  |  |  |  |  |  |  | 0.09 | ± | 0.07 |
|  | 31 | 0.01 | ± | 0.01 |  |  |  |  |  |  |  |  |  | 0.01 | ± | 0.00 |
|  | 32 | 0.02 | ± | 0.02 |  |  |  |  |  |  |  |  |  | 0.03 | ± | 0.03 |
|  | 34 |  |  |  |  |  |  |  |  |  |  |  |  |  |  |  |
|  |  |  |  |  |  |  |  |  |  |  |  |  |  |  |  |  |
| Ketone | 29 |  |  |  |  |  |  |  |  |  |  |  |  | 0.02 | ± | 0.01 |
|  | 31 |  |  |  |  |  |  |  |  |  |  |  |  | 0.02 | ± | 0.00 |
|  |  |  |  |  |  |  |  |  |  |  |  |  |  |  |  |  |
| *n*-alkanes | 22 |  |  |  |  |  |  |  |  | 0.01 | ± | 0.00 |  |  |  |  |
|  | 23 |  |  |  |  | 0.18 | ± | 0.09 |  | 0.01 | ± | 0.00 |  | 0.01 | ± | 0.00 |
|  | 25 | 0.20 | ± | 0.21 |  | 0.57 | ± | 0.07 |  | 0.04 | ± | 0.00 |  | 0.04 | ± | 0.01 |
|  | 26 | 0.05 | ± | 0.06 |  | 0.08 | ± | 0.01 |  | 0.03 | ± | 0.01 |  | 0.03 | ± | 0.01 |
|  | 27 | 1.05 | ± | 1.14 |  | 1.40 | ± | 0.21 |  | 0.08 | ± | 0.01 |  | 0.06 | ± | 0.01 |
|  | 28 | 0.30 | ± | 0.31 |  | 0.15 | ± | 0.02 |  | 0.05 | ± | 0.01 |  | 0.04 | ± | 0.01 |
|  | 29 | 4.67 | ± | 4.77 |  | 1.21 | ± | 0.19 |  | 0.18 | ± | 0.06 |  | 0.15 | ± | 0.03 |
|  | 30 | 0.14 | ± | 0.12 |  | 0.04 | ± | 0.01 |  | 0.05 | ± | 0.01 |  | 0.06 | ± | 0.01 |
|  | 31 | 0.06 | ± | 0.06 |  | 0.02 | ± | 0.00 |  | 0.22 | ± | 0.06 |  | 0.10 | ± | 0.02 |
|  | 32 |  |  |  |  |  |  |  |  |  |  |  |  | 0.01 | ± | 0.00 |
|  | 33 | 0.01 | ± | 0.01 |  |  |  |  |  |  |  |  |  | 0.01 | ± | 0.00 |
|  |  |  |  |  |  |  |  |  |  |  |  |  |  |  |  |  |
| *iso*-alkane | 25 |  |  |  |  | 0.06 | ± | 0.05 |  |  |  |  |  |  |  |  |
|  | 27 | trace |  |  |  | 0.03 | ± | 0.01 |  |  |  |  |  | trace |  |  |
|  |  |  |  |  |  |  |  |  |  |  |  |  |  |  |  |  |
|  |  |  |  |  |  |  |  |  |  |  |  |  |  |  |  |  |
| *n*-alkenes | 25 |  |  |  |  | 0.03 | ± | 0.01 |  |  |  |  |  |  |  |  |
|  | 26 |  |  |  |  |  |  |  |  |  |  |  |  | trace |  |  |
|  | 27 | 0.03 | ± | 0.03 |  | 0.02 | ± | 0.01 |  |  |  |  |  | trace |  |  |
|  | 28 |  |  |  |  |  |  |  |  |  |  |  |  | 0.02 | ± | 0.00 |
|  | 29 | 0.22 | ± | 0.23 |  | 0.04 | ± | 0.01 |  |  |  |  |  |  |  |  |
|  | 31 | 0.21 | ± | 0.23 |  |  |  |  |  |  |  |  |  |  |  |  |
|  |  |  |  |  |  |  |  |  |  |  |  |  |  |  |  |  |
| Alkyl esters | 39 |  |  |  |  | 0.01 | ± | 0.01 |  |  |  |  |  |  |  |  |
|  | 41 |  |  |  |  | 0.01 | ± | 0.01 |  |  |  |  |  |  |  |  |
|  | 42 | 0.01 | ± | 0.01 |  | 0.05 | ± | 0.01 |  |  |  |  |  |  |  |  |
|  | 43 |  |  |  |  | 0.03 | ± | 0.02 |  |  |  |  |  |  |  |  |
|  | 44 | 0.03 | ± | 0.03 |  | 0.15 | ± | 0.05 |  |  |  |  |  |  |  |  |
|  | 45 |  |  |  |  | 0.04 | ± | 0.01 |  |  |  |  |  |  |  |  |
|  | 46 | 0.03 | ± | 0.03 |  | 0.16 | ± | 0.03 |  |  |  |  |  |  |  |  |
|  |  |  |  |  |  |  |  |  |  |  |  |  |  |  |  |  |
| not identified |  | 1.32 | ± | 0.54 |  | 2.11 | ± | 0.32 |  | 0.21 | ± | 0.07 |  | 0.26 | ± | 0.17 |
|  |  |  |  |  |  |  |  |  |  |  |  |  |  |  |  |  |
| sum of acyclic components |  | 7.64 | ± | 1.30 |  | 5.29 | ± | 0.62 |  | 0.83 | ± | 0.17 |  | 1.26 | ± | 0.47 |
|  |  |  |  |  |  |  |  |  |  |  |  |  |  |  |  |  |
| total wax |  | 8.95 | ± | 1.68 |  | 7.41 | ± | 0.85 |  | 1.04 | ± | 0.20 |  | 1.52 | ± | 0.51 |







(B)

(A)

Fig. S1 SEM observation of the surface of lily tepals at (A) inner surface and (B) outer surface. Stomata are observed at outer tepal- but not at inner tepal surfaces.

Fig. S2 Drying curve of a representative leaf of Lily cv. ‘Tiber’ at 20°C. The leaf conductance was plotted against the relative water deficit (RWD). The initial conductance was very high, after reach a certain RWD (= 0.05), the change of conductance was constant and linear. The transition point indicates the point of maximum stomatal closure for dehydration, which help to indicate the cuticular transpiration. The mini conductance of leaf was the mean of the water permeance excluding the initial two value.

Fig. S3 Drying curve (A to D) and weight loss curve (E to H) of a representative tepal of Lily cv. ‘Casa blanca’ at 20°C. The water permeance was plotted against the relative water deficit (RWD). The weight was against the desiccation time. A and E, B and F, C and G, D and H represent the inner tepal of green bud, outer tepal of green bud, inner tepal of open flower and outer tepal of open flower, respectively.

Fig. S4 Drying curve (left) and weight loss curve (right) of a representative inner tepal of Lily cv. ‘Tiber’ at 20°C. The water permeance was plotted against the relative water deficit (RWD). The weight was against the desiccation time.
